# When rare diseases crisscross within the same patient: von Hippel-Lindau and type 1 gastric neuroendocrine tumor

**DOI:** 10.1007/s42000-024-00556-9

**Published:** 2024-04-15

**Authors:** Krystallenia I. Alexandraki, Ariadni Spyroglou, Paraskevi Xekouki, Konstantinos I. Bramis, Georgios Kyriakopoulos, Konstantinos Barkas, Ioannis S. Papanikolaou, George Mastorakos, Manousos Konstadoulakis

**Affiliations:** 1 2nd Department of Surgery, Medical School, National and Kapodistrian University of Athens, Athens, Greece; 2 Endocrinology and Diabetes Clinic, University Hospital of Heraklion, University of Crete School of Medicine, Voutes, Heraklion Crete, Greece; 3 Department of Pathology, Evangelismos Hospital, Athens, Greece; 4 Department of Neurosurgery, General Hospital of Nikaia-Peiraia, Agios Panteleimon, Athens, Greece; 5 Hepatogastroenterology Unit, Second Department of Internal Medicine-Propaedeutic, National and Kapodistrian University of Athens, “Attikon” University General Hospital, Athens, Greece

**Keywords:** Von-Hippel-Lindau, Gastric neuroendocrine neoplasm, Chronic autoimmune gastritis, Clear cells

## Abstract

Von-Hippel-Lindau (VHL) is a genetic multisystem disorder characterized by visceral cysts and benign and malignant tumors in various organs. Herein, we present the case of a 23-year-old woman with VHL presenting with multiple gastric neuroendocrine neoplasms (gNENs) type 1 in the context of chronic autoimmune gastritis (CAG). Although gNENs are not acknowledged as a typical entity in VHL patients, in the present case, gNENs were composed of neoplastic cells with clear cytoplasm usually seen in tumors related to VHL disease. We additionally performed a literature review on the presence of neuroendocrine clear cell tumors and report on further cases of clear cell NENs. The present case illustrates that clear-cell transformation in gNENs may be due to the dual genetic background of the patient; the real oncogenic stimulus may be more closely related to CAG than to VHL disease accompanied by an interplay between neoplastic and autoimmune processes. Therefore, close monitoring of patients with clear cell NENs appears to be important before excluding VHL disease, even in the context of phenotypically unrelated diseases.

## Introduction

Von Hippel-Lindau (VHL) disease is an autosomal dominantly inherited multiple tumor syndrome resulting from a germline mutation in the VHL gene (located on chromosome 3). Its incidence rate is 1 per 36,000 live births. VHL disease can lead, in various organ systems, to the development of benign and malignant tumors, such as hemangioblastomas in the central nervous system (CNS) and retina, renal cysts, and clear cell renal cell carcinomas, pheochromocytomas, pancreatic cysts, and pancreatic neuroendocrine neoplasms (panNENs) [1]. The surveillance protocol recommended in adults with VHL includes annual retinal inspection, annual neurological examination, annual plasma metanephrines, plasma normetanephrines and plasma chromogranin A (CgA) measurement, annual ultrasound/magnetic resonance tomography (MRI) of the abdomen (alternately), and every 2 years MRI of the CNS, including the inner ear [2].

In the context of VHL pathophysiology, when oxygen is present in the cell, the VHL protein is responsible for the ubiquitination and, thus, the degradation of hypoxia inducible factor 1 a (HIF1a); however, in hypoxia or due to VHL mutations, HIF1a remains active and stimulates various growth factors inducing tumorigenesis [3]. VHL tumorigenesis conforms to the “two-hit-model” where the first hit, that is the germline VHL mutation, exists already at birth, while the second hit, that is the somatic VHL mutation of the second allele, is the trigger for tumor development in the respective organ [1].

On the other hand, gastroenteropancreatic NENs are still considered rare diseases despite the increase of their incidence in recent years [4]. To quote one example, the incidence of gastric neuroendocrine neoplasms (gNENs) increased from 7- to 10-fold during recent years [5]. The pathogenesis of type 1 gNENs is closely related to autoimmunity and relies on the presence of circulating anti-parietal cell antibodies (APCA) which attack parietal cells, causing chronic autoimmune gastritis (CAG) and, thus, leading to an insufficient reduction of the gastric pH, which, in turn, stimulates gastrin secretion from the G-cells and, thereby, hyperplasia/dysplasia of the enterochromaffin-like (ECL) cells responsible for the formation of gNENs. The prevalence of gNENs in CAG patients varies from 1.5–12.5% and the vast majority are diagnosed incidentally following upper gastrointestinal endoscopy (UGIE) during investigation of dyspepsia and/or anemia [5, 6]. Type 1 gNENs are usually multiple, presenting as polyps of the gastric body, with a size of < 1–2 cm, characterized by a low ki-67 index (< 2%); they metastasize rarely and can be either followed up or excised endoscopically and, very occasionally, surgically [6].

## Case report

A 23-year-old woman presented to our clinic to obtain advice concerning a gastric lesion. Her medical history included a recent episode of intracerebral hemorrhage 18 months previously, followed by coma, which was resolved after arterial embolization. Diagnosis of VHL was suspected 6 months later following resection of two hemangioblastomas, one from the cerebellum and one from the cervical spinal cord. Diagnosis was confirmed by genetic analysis showing a c.484 T > C/p.(Cys162Arg) mutation of the VHL gene. Her family history revealed that her mother had suffered from quadriparesis due to endolymphatic sac tumors; she deceased when the patient was still very young. Following diagnosis of VHL disease in the patient, three retinal hemangioblastomas were revealed and subsequently removed. On direct questioning for unrelated concomitant medical conditions, she reported a recent diagnosis of Graves’ disease (GD) treated with carbimazole.

**Fig. 1:**
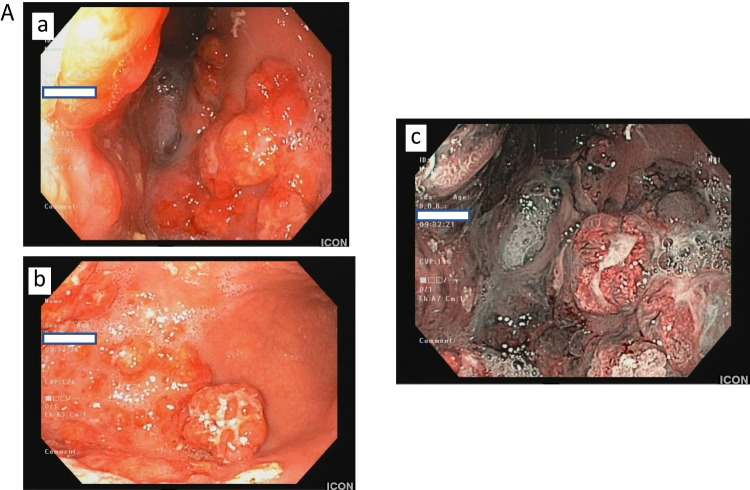
Upper gastrointestinal endoscopy demonstrating several gastric NENs of various sizes, ranging between 0.8 and 2.5 cm (**a**). Note the extensive atrophy of the “normal” gastric mucosa next to the lesions (**b**). The same features are also illustrated using high-definition combined with narrow-band imaging (**c**)

**Fig. 2:**
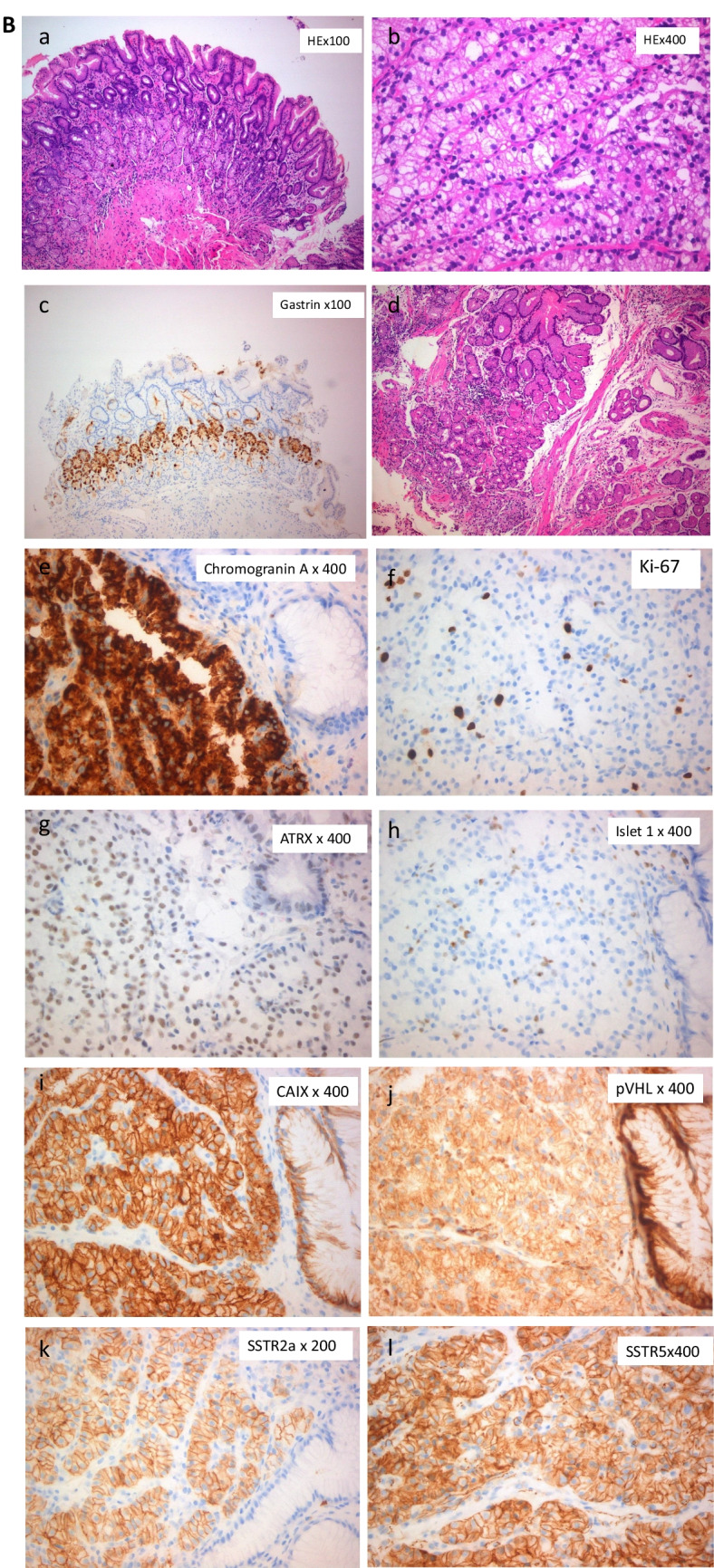
Pyloric type gastric mucosa with mild reactive gastropathy. Mild hyperplasia of G cells of the antrum (**a**, **b**: hematoxylin and eosin staining; **c**: gastrin). The tumors were composed of clear cells arranged in nests and tubules separated by a delicate capillary sized vascular network. They had clear cytoplasm containing small vacuoles and round nuclei with finely powdery chromatin and inconspicuous nucleoli. Rare cells with cytological atypia were identified (**d**). Immunostaining for neuroendocrine markers (chromogranin A (**e**)), Ki-67 proliferation index5% (**f**), transcription factors for neuroendocrine neoplasms origin (ATRX (**g**), islet-1 (**h**), strong membranous expression of CA-IX (**i**), mild cytoplasmic and membranous expression of pVHL(**j**), and somatostatin receptors (SSTR) which both showed strong and complete membranous expression (SSTR2a (**k**), SSTR5 (**l**))

During VHL disease monitoring, the MRI of the abdomen showed four pancreatic cysts and a thickened gastric corpus, this leading to an indication for UGIE. An UGIE was performed in a regional hospital where several gastric polyps of the entire corpus of the stomach were documented and a suspicion of the presence of hemangioblastomas or a gastric adenocarcinoma was raised. In view of the patient’s history of hemangioblastoma, biopsies were limited. Nevertheless, taking into consideration the documented autoimmune thyroid disease, that is, GD [7], the suspicion for a gNEN was raised. An UGIE was repeated in a specialized center for NENs in accordance with the guidelines’ suggestions for the assessment of representative biopsies not only from the tumor but also from the surrounding mucosa and other parts of the stomach (antrum and body-fundus) in order to provide a wider image of the nature of the specific lesion. Repeat UGIE showed several gastric NENs of different sizes, the largest measuring 2.5 cm. In the meantime, proton pump inhibitor (PPI) treatment was discontinued and serum gastrin and CgA levels were found to be increased [1295 pg/ml (< 110) and 540 ng/ml (< 120), respectively]. Gastric biopsy showed a multiplication index, Ki-67 of 5% with a mitotic index 0.4 over 10 high-power fields (HPF), while neoplasms infiltrated the muscularis mucosa (Fig. 1). Neoplasmatic foci, documented in 8/13 biopsies, consisted of medium-sized cells with uniform round to oval, often peripherally located nuclei with finely granular chromatin and abundant eosinophil and clear cytoplasm. Moreover, an extensive immunohistochemical analysis for neuroendocrine markers and transcriptional factors was as follows: positive CgA and synaptophysin in 100 and 90% of the neoplastic cells, respectively; cytokeratin 8/18 in 100% of the neoplastic cells; PDX1 (pancreatic and duodenal homeobox 1) in 35% of the neoplastic cells (mild nuclear staining); islet-1 (moderate nuclear staining); and somatostatin receptors type 2a and type 5 both showing strong and complete membranous expression (score 3 according to the Volante score). On the other hand, staining for CDX-2 (caudal-type homeobox transcription factor 2), gastrin, somatostatin, and serotonin were negative (Fig. 2). ATRX expression (alpha-thalassemia/mental retardation syndrome X-linked) was retained; menin was strongly expressed suggesting no underlying mutation in the context of multiple endocrine neoplasia syndrome type 1. In the context of VHL, VHL protein demonstrated mild-to-moderate granular cytoplasmatic staining in 100% of the neoplastic cells and carbonic anhydrase IX (CA-IX) membranous but not cytoplasmic staining pattern (Fig. 2). Clear cell morphology was in accordance with the membranous expression of CA-IX, indicating a malfunction of the pseudohypoxia signaling pathway. Neoplastic cells with eosinophilic and non-clear cytoplasm were observed in a few lesions smaller in diameter, or they were intermixed in the larger lesions with the cells with clear cell morphology. The gastric mucosa of the corpus was characterized by extensive atrophy with extensive pseudopyloric metaplasia, moderate chronic inflammation, and linear and micronodular hyperplasia of ECL cells. The mucosa of the antrum was characterized by reactive gastropathy and mild hyperplasia of the gastrin-producing neuroendocrine cells (G-cells). Helicobacter pylori bacteria were not found in any of the two types of gastric mucosa.

On the 68-Ga DOTATATE PET/CT, a mildly increased uptake of the tracer was documented in the gastric antrum (SUVmax 8.1). The presence of uncommonly extensive gNEN [8] in such a young patient was discussed in detail during our multidisciplinary tumor board. Since somatostatin-analogue treatment (SSAT) reduces gastrin production, resulting in suppression of the stimulus for further tumor growth and progression, it was offered to this patient, in accordance with the recent guidelines in the case of multiple large tumors [9], despite the fact that recurrence should be anticipated post-SSAT in type 1 gNENs [10]. Overall, taking into consideration the young age of the patient (23 years old at the time of diagnosis), the radical character of the surgical treatment of this condition, and the hesitation of the patient, it was decided to offer her the alternative of being treated with conservative medical treatment followed by re-evaluation of the findings with close follow-up, which the patient agreed to. Thus, octreotide long-acting repeatable (LAR) 30 mg every 28 days intramuscularly was initiated, followed by lanreotide autogel 120 mg subcutaneously because of a mild allergic reaction to the first compound. In the follow-up UGIE 1 year later, the number of the smaller gNENs appeared slightly improved, as measured by the gastroenterologist. However, as the lesions were in some parts of the stomach almost confluent, no direct comparisons of the numbers pre- and post-SSAT could be made and this initial macroscopic impression could not be proved by the dimensions of the individual gNENs. The histological analysis documented well-differentiated gNENs G2 (WHO 2019), with a ki-67 of 7%. As no improvement could be detected after 1 year of SSAT, a total gastrectomy was proposed by the specialized tumor board, and the patient consented. The intervention was performed with an uncomplicated postoperative course. In the histological examination, numerous polypoid tumors with a size range from 0.5–2.5 cm, in part extending into the submucosa and in part with ulceration (pT2m), were identified. Lesions extended starting approximately 3 cm from the lower esophageal sphincter up to the prepyloric area (two lesions). Microscopically, numerous well-differentiated gNENs grade 1, with a ki-67 of 2–2.46%, and 0.4 mitoses/10 HPF were found. To date, 12 months post-surgery, the patient has remained clinically stable and no further abnormalities were located in the latest biochemical and imaging follow-up tests for the VHL. Both the final histology and the follow-up of the patient confirmed our first impression of a primary gastric neoplasm rather than a possible pancreatic secondary focus. The immunohistochemical assessment, which was performed with the transcription factors PAX-6, CDX-2, PDX-1, and ISLET-1, did not support the pancreatic origin. Moreover, according to the initial MRI report, 3–4 small cystic lesions of a few millimeters in diameter were documented in the pancreatic corpus, which, at a 4-year follow-up visit, were seen to have remained stable in number and size.

## Review of the literature and discussion

To date, the presence of a gNEN in a patient with VHL has, to the best of our knowledge, only been described in a recent report by Kawaguchi et al., where a 45-year-old VHL patient presented with multiple gastric polyps, histologically diagnosed as NENs grade 2. Histologically, tumor cells were described with a circular nucleus and abundant foamy or granular cytoplasm. However, in this case, no atrophy of the gastric mucosa was observed, APCA antibodies were negative, while gastrin levels were slightly elevated, it not being clear whether this increase was due to PPI treatment. Interestingly, in the above publication, the discontinuation of PPI treatment resulted in a pronounced regression of these gNENs. Thus, the authors attributed the gNEN development to the chronic ECL stimulation from increased gastrin levels due to the PPI administration [11].

To further elucidate the presence of clear cells in NENs in VHL patients, a literature search with the keywords “von hippel lindau,” “neuroendocrine tumor,” and “clear cell” (von hippel[Author] AND ("lindau"[All Fields] OR "lindau s"[All Fields]) AND ("neuroendocrine tumor"[All Fields] OR "neuroendocrine tumors"[MeSH Terms] OR ("neuroendocrine"[All Fields] AND "tumors"[All Fields]) OR "neuroendocrine tumors"[All Fields] OR ("neuroendocrine"[All Fields] AND "tumor"[All Fields]) OR "neuroendocrine tumor"[All Fields]) AND ("clear"[All Fields] OR "cleared"[All Fields] OR "clearing"[All Fields] OR "clearings"[All Fields] OR "clears"[All Fields]) AND ("cells"[MeSH Terms] OR "cells"[All Fields] OR "cell"[All Fields]) was performed. Only four articles could be retrieved following the search, out of which two had to be excluded due to irrelevant content. From the citations of one publication, two further articles could be included in the analysis.

Specifically, Woo et al. described the case of a 47-year-old VHL patient presenting with a pancreatic lesion and two concomitant renal lesions. While the renal lesions were diagnosed as a clear cell and a cystic renal cell carcinoma, respectively, the pancreatic lesion was a well-circumscribed yellow solid mass, entirely consisting of clear cells. Although the initial assumption was that this lesion was a metastasis from the renal carcinoma, immunohistochemical analysis positive for the neuroendocrine markers and vimentin, classified the pancreatic lesion as a clear cell pancreatic neuroendocrine tumor [12]. Hoang et al. reported on five cases of clear cell panNENs in VHL patients, all of which presented histologically with a component of clear cells arranged in nests. Here again, two of the tumors were initially confused with metastatic renal cell carcinoma [13]. Similarly, Sinkre et al. described the case of a 38-year-old man with VHL disease, presenting with a carcinoid tumor of the gallbladder with lipid-containing clear cells, also initially interpreted as renal cell carcinoma, although it was positive for CgA, synaptophysin, cytoceratins, and inhibin [14]. Finally, Gucer et al. reported on a 60-year-old woman with known VHL and pancreatic lesions, positive on somatostatin receptor scintigraphy (OctreoScan), who underwent Whipple resection. Histologically, the resected pancreatic lesions were identified as multiple grade 2 NENs with variable clear cell change. In parallel, an incidental tubular adenoma with low-grade dysplasia was found in the ampulla and, adjacent to this lesion, an epithelial neoplasm in the duodenal ampullary mucosa was identified. This lesion consisted of epithelial cells resembling lipoblasts or signet ring cells, positive for inhibin [15].

In line with the previously reported cases, the gNENs identified in our patient were also composed of neoplastic cells with clear cytoplasm (clear cells), usually seen in tumors related to VHL disease [16]. The clear cell genotype–phenotype correlation has been observed in VHL disease and in VHL-associated tumors. The clear cell phenotype has been attributed to the activation of HIF1a resulting in a status of pseudohypoxia and, consequently, in lipid and glycogen accumulation within the tumor cells.

Interplay of neoplastic and autoimmune processes has recently been reported [17]. Despite the fact that these processes were initially thought to have opposite immunological properties, it has been proposed that cancer progresses from a proinflammatory state to an anti-inflammatory one, whereas an autoimmune disease can ab initio be characterized a proinflammatory state [17]. The mechanism that leads to their combined presence is based on a temporal change of immune response during the course of cancer development. Consequently, the present case illustrates that the histological finding of the change of clear cells may be dependent on the genetic background of VHL, while the real oncogenic stimulus can be more closely related to CAG than to the VHL disease. Therefore, it appears important to perform further clinical, biochemical, and imaging screening in patients presenting with clear cell NENs before excluding VHL disease [16].

The coexistence of autoimmune diseases in the context of neoplasmatic disease has been described as paraneoplastic syndrome; inversely, neoplasmatic disease has also been described in the context of immunosuppressive treatment. Moreover, it has been suggested that the proinflammatory environment of autoimmune traits may support both initiation and growth of early malignancy; hence, the low-grade inflammation of the autoimmune disease may prolong survival in a neoplasm with a low-malignant potential as opposed to more aggressive neoplasms that are early on characterized by increased immunological evasion [17]. Although a similar interplay between neoplastic and autoimmune processes can be hypothesized in this case report, a clear link between clear cell NENs and autoimmunity against a VHL background cannot easily be substantiated, as such cases are quite rare. A previous study of a case of VHL disease and another autoimmune disease, myasthenia gravis, investigated the role of the HIF-1α pathway and hypoxia in inflammatory response, underlining the need for further insight into the clinical, genetic, and molecular overlap of neoplasia and autoimmunity [18]. Thus, the establishment of an international multicenter registry for such patients would enable further observations and clinical, genetic, molecular, and histological correlations to corroborate this initial finding.

In conclusion, the current case illustrates that histopathological features should be considered and investigated separately, since more than one pathogenetic mechanism may crisscross within the same patient, also affecting the patient’s prognosis.
